# Propuesta de manejo inicial del infarto de miocardio con elevación del segmento ST no complicado en centros sin capacidad de intervención coronaria percutánea en el Perú

**DOI:** 10.47487/apcyccv.v4i4.335

**Published:** 2023-12-27

**Authors:** Piero Custodio-Sánchez, David Miranda-Noé, L. Marco López-Rojas, Cynthia Paola Paredes Paucar, W. Germán Yábar Galindo, Paol Rojas De La Cuba, Jorge Orlando Martos Salcedo, Manuel Chacón-Diaz

**Affiliations:** 1 Unidad de Cardiología Intervencionista, Hospital Nacional Almanzor Aguinaga Asenjo, Chiclayo, Perú. Unidad de Cardiología Intervencionista Hospital Nacional Almanzor Aguinaga Asenjo Chiclayo Perú; 2 Servicio de Cardiología Clínica. Instituto Nacional Cardiovascular INCOR, Lima, Perú. Servicio de Cardiología Clínica Instituto Nacional Cardiovascular INCOR Lima Perú; 3 Hospital Nacional Hipólito Unanue, Lima, Perú. Hospital Nacional Hipólito Unanue Lima Perú; 4 Unidad de insuficiencia cardiaca, Hospital Germans Trias i Pujol, Barcelona, España. Unidad de insuficiencia cardiaca Hospital Germans Trias i Pujol Barcelona España; 5 Hospital Nacional Guillermo Almenara Irigoyen, Lima, Perú. Hospital Nacional Guillermo Almenara Irigoyen Lima Perú; 6 Servicio de Cardiología. Hospital Regional Docente de Cajamarca, Cajamarca, Perú. Servicio de Cardiología Hospital Regional Docente de Cajamarca Cajamarca Perú; 7 Unidad Cardiovascular. Clínica Delgado AUNA, Lima, Perú. Unidad Cardiovascular Clínica Delgado AUNA Lima Perú

**Keywords:** Infarto del Miocardio con elevación del ST, Fibrinolisis, Perú, Redes de Atención, ST Elevation Myocardial Infarction, Fibrinolysis, Peru, Community Networks

## Abstract

El infarto de miocardio con elevación del segmento ST (IMCEST) es una entidad clínica cuyo tratamiento adecuado dependerá de su pronto reconocimiento, diagnóstico certero y manejo en redes de reperfusión. El primer contacto con estos pacientes es generalmente en centros sin capacidad de reperfusión, atendidos por médicos no cardiólogos y en centros alejados de hospitales de mayor capacidad resolutiva, algo que es bien conocido en nuestro país. Este manuscrito propone la estrategia de diagnóstico y tratamiento del IMCEST para centros sin capacidad de intervención coronaria percutánea del sistema público de salud en el Perú, haciendo hincapié en no perder de vista patrones electrocardiográficos compatibles con oclusión de la arteria coronaria, fibrinolisis adecuada y manejo de sus complicaciones, el tratamiento del infarto en poblaciones especiales y resaltando la importancia de la estrategia farmacoinvasiva como forma de tratamiento de reperfusión principal en nuestro país.

## Abstract (en)

ST-segment elevation myocardial infarction (STEMI) is a clinical entity whose adequate treatment will depend on its prompt recognition, accurate diagnosis, and management in reperfusion networks. The first contact with these patients is generally done in centers without reperfusion capacity, attended by non-cardiologist doctors, and in centers far from hospitals with greater resolution capacity, something that is well known in our country. This manuscript proposes a strategy for the diagnosis and treatment of STEMI in centers without percutaneous coronary intervention capacity of the public health system in Peru, emphasizing not losing sight of electrocardiographic patterns compatible with coronary artery occlusion, adequate fibrinolysis and management of its complications, the treatment of infarction in special populations and highlighting the importance of the pharmacoinvasive strategy as the main form of reperfusion treatment in our country.

## Introducción

El infarto de miocardio con elevación del segmento ST (IMCEST), representa una emergencia médica cuya resolución dependerá de un diagnóstico y tratamiento adecuado y, sobre todo, oportuno. Pocos centros en nuestro país cuentan con cardiólogos de guardia permanentemente en las emergencias hospitalarias, por lo que el objetivo de este artículo es llevar un mensaje a todo el personal de salud que enfrenta el IMCEST en el primer nivel de contacto con el paciente, con la finalidad de sistematizar los pasos del diagnóstico oportuno tanto clínico como electrocardiográfico, evaluar las características particulares de los centros hospitalarios (Ministerio de Salud, EsSalud, de la capital y del interior del país) en cuanto a los tiempos para referir a un paciente para intervencionismo coronario, y una puesta al día en la terapia fibrinolítica paso a paso, con algunas consideraciones en poblaciones especiales a tener en cuenta.

## Diagnóstico precoz

### Presentación clínica

El síntoma más frecuente que pone en marcha el proceso diagnóstico y terapéutico de los pacientes con sospecha de síndrome coronario agudo (SCA) es el dolor torácico. La sospecha inicial de infarto agudo de miocardio parte del contexto clínico (características del paciente, características del dolor incluyendo su temporalidad y el examen físico) ^1^^,^^2^, a lo cual se sumará los exámenes auxiliares que confirmarán nuestra sospecha.

El dolor torácico anterior agudo junto a otros parámetros semiológicos, que presenta una escala de Geleijnse modificada >= 6 puntos, indica un origen isquémico agudo con alta sensibilidad ^3^^,^^4^. Las otras características semiológicas que aumentan la probabilidad de un origen isquémico del dolor torácico son: el dolor central (por ejemplo, signo de Levine), difuso, tipo opresión, compresión, constricción, pesadez, opresivo, relacionado con el ejercicio/estrés, retroesternal, que alivia parcial o totalmente con el reposo y/o el uso de nitratos tiene mayor probabilidad de tener un origen isquémico ^5^.

La valoración clínica debe tomar en cuenta los factores de riesgo individuales. La presencia de factores de riesgo para ateroesclerosis (sexo, edad, diabetes *mellitus*, hipertensión arterial, tabaquismo, dislipidemia, enfermedad renal crónica, entre otros) aumenta las probabilidades de que el dolor torácico sea debido a isquemia miocárdica ^2^. En pacientes mujeres, los ancianos, diabéticos, enfermedad renal crónica o con demencia, se deben tener en cuenta la presencia de síntomas atípicos como: disnea, síncope, alteración del estado de conciencia, diaforesis, náuseas, vómitos, dolor epigástrico, debilidad, mareo, inestabilidad a los cuales denominaremos equivalentes anginosos y serán a veces el motivo por el cual estos pacientes se presenten a urgencias ^2^.

En la evaluación inicial de un paciente con sospecha de SCA se debe realizar una adecuada historia clínica, preguntando por antecedentes y factores de riesgo cardiovasculares, características, hora de inicio y duración del dolor torácico, además de otros síntomas acompañantes (neurovegetativos o equivalentes anginosos). Se debe valorar en el examen físico signos de insuficiencia cardiaca (estertores en campos pulmonares, tiraje, la presencia de tercer ruido, soplos, ingurgitación yugular, entre otros). Finalmente, es importante resaltar que ninguna característica clínica aislada es suficiente para excluir o confirmar un SCA, por lo que, en presencia de un paciente con dolor torácico (sin causa no cardiaca evidente) y/o sospecha clínica de isquemia aguda, lo adecuado es tomar inmediatamente un electrocardiograma ^2^^,^^5^.

### El electrocardiograma (EKG)

El objetivo de tiempo máximo entre el primer contacto médico (PCM) y la toma e interpretación del electrocardiograma deber ser menor o igual a 10 min ^5^.

El PCM es el momento de la primera evaluación del paciente realizada por un médico, personal paramédico u otro personal de urgencias, con capacidad para obtener e interpretar el EKG y proporcionar intervenciones iniciales. El PCM puede ocurrir en un contexto prehospitalario o a la llegada del paciente al hospital.

**Figura 1:**
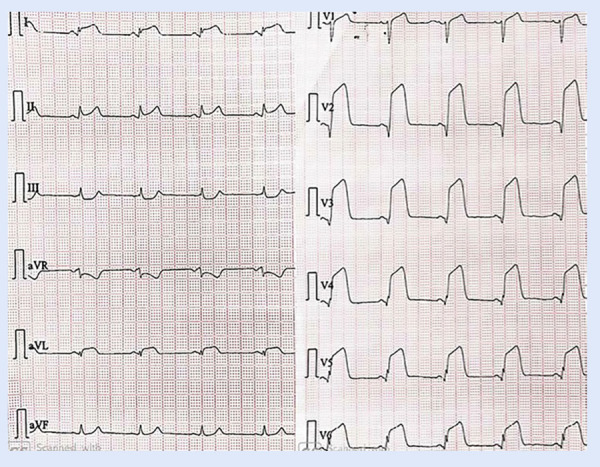
Electrocardiograma de paciente con infarto de miocar dio anterior extenso que muestra elevación del ST en derivaciones V2 a V6, DI y aVL, y depresión ligera recíproca del ST en derivaciones III y aVF.

El SCA ST elevado se define como la presencia de síntomas de isquemia miocárdica aunado a la presencia en el electrocardiograma de un punto «J» elevado mayor o igual de 1 mm en dos o más derivadas contiguas en la misma cara y en ausencia de hipertrofia ventricular izquierda o bloqueo de rama izquierda **(**figura 1**)**. En las derivadas V2 a V3 se deberá tomar un punto de corte mayor dependiendo del sexo y la edad **(**Tabla 1**)**. Debe realizarse la toma de las derivadas V7, V8 y V9 en pacientes con sospecha de un infarto posterior, y las derivadas derechas V3R y V4R en pacientes con un infarto inferior para identificar el infarto del ventrículo derecho concomitante **(**Tabla 2**)** ^(^^6^.

**Tabla 1:** Punto de corte, según sexo y edad, en las derivadas V2 a V3 para definir elevación significativa del segmento ST ^(^^6^

Sexo	Derivada V2 - V3	Otras derivadas
Femenino	≥ 1,5 mm	
Masculino ≥ 40 años	≥ 2 mm	≥ 1 mm
Masculino < 40 años	≥ 2,5 mm	

**Tabla 2:** Presentaciones electrocardiográficas atípicas ^(^^6^

Territorio	Derivadas	Segmento ST	Magnitud	Otros cambios del ECG
Infero-Basal	V1 a V3	Depresión	≥ 1 mm	Onda R alta en V1 - V2, con relación R/S > 1 en V2. Onda T positiva en V1 a V3
	V7 a V9	Elevación	≥ 0,5 mm	En varones < 40 años, la elevación es ≥ 1 mm.
Tronco común izquierdo o multivaso*	AVR	Elevación	≥ 1 mm	Asociado a depresión del ST en ≥ 8 derivadas.
Ventrículo derecho	V3R y V4R	Elevación	≥ 0,5 mm	En varones < 30 años la elevación es ≥ 1 mm. Elevación del ST en V1 ≥ 1 mm.

* Presentación electrocardiográfica que indica obstrucción del tronco común (o equivalente), o isquemia multivaso especialmente si el paciente presenta deterioro hemodinámico, se sugiere considerar una intervención coronaria percutánea primaria inmediata en estos casos.

Hay que considerar que la isquemia miocárdica se asocia con cambios dinámicos en el EKG, la realización de EKG seriados (cada 15-30 min) puede aportar información, sobre todo cuando el EKG inicial no es diagnóstico; además, cambios recíprocos en las derivadas opuestas pueden ayudar a diferenciar el IMCEST de la pericarditis o patrones de repolarización precoz donde no se verá este hallazgo ^(^^6^.

**Figura 2:**
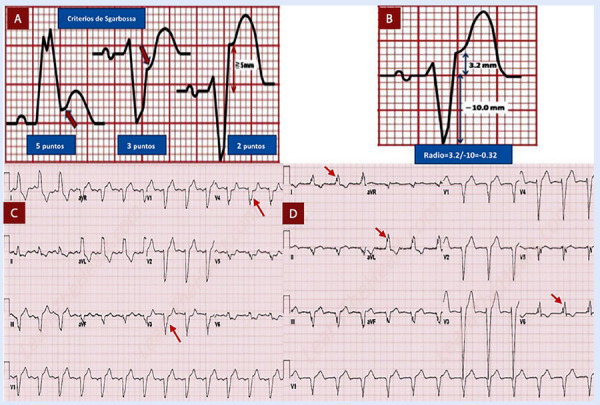
Representación esquemática de diferentes criterios electrocardiográficos para diagnóstico de infarto en presen cia de bloqueo de rama izquierda del Has de His. **A)** Criterios de Sgarbossa; **B)** Criterio de Smith o Sgarbossa modificado; **C)** Criterio de Cabrera - Friedland. Las flechas rojas muestran una muesca prominente de > 50ms de duración en la rama ascendente de la onda S de V3 a V4. **D)**Criterio de Chapman-Pearce. Las flechas rojas muestran una melladura prominente en la rama ascendente de la onda R en DI, aVL y V6 ^7^.

En la cronología de los cambios EKG, debe tenerse en cuenta que primero aparecerán ondas T hiperagudas que preceden a la elevación del segmento ST. Un signo característico de isquemia grave es el signo de aleta de tiburón formado por la fusión del QRS, ST y ondas T (amplitud >= 10 mm) ^6^. En el contexto de bloqueo de rama izquierda o ritmo de marcapaso se debe utilizar los criterios de Sgarbossa, un punto de corte >=3 puntos indica IMCEST con una alta sensibilidad y especificidad **(**Figura 2**y**3**)**^7^^,^^8^.

**Figura 3:**
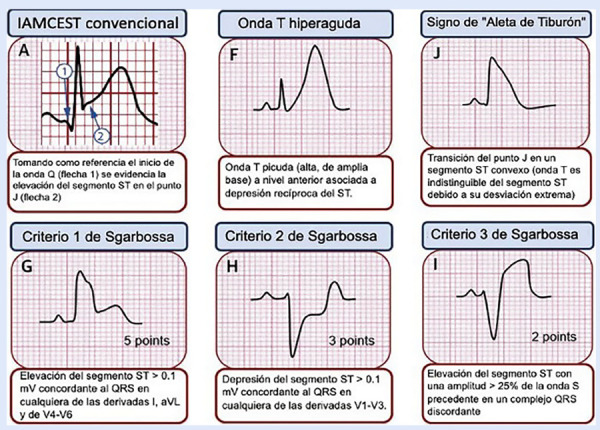
Patrones electrocardiográficos que pueden presentarse en el paciente con infarto de miocardio ST elevado. Modificado de Asatryan B. y col.^8^.

## Reperfusión: situación actual y propuesta de manejo

### Realidad Nacional

**Figura 4:**
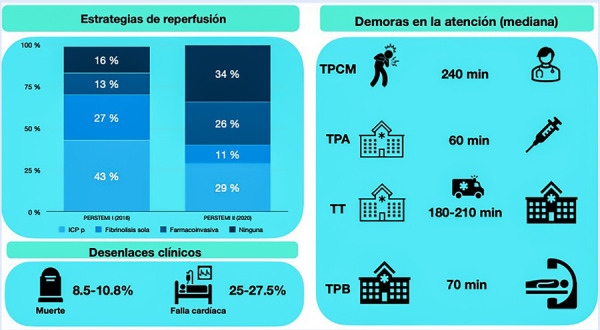
Realidad nacional del tratamiento del infarto de miocardio con elevación del segmento ST. ICPp= intervención coronaria percutánea primaria. TPCM=tiempo a primer contacto médico. TPA= tiempo puerta-aguja. TT= tiempo de trans ferencia interhospitalaria. TPB= tiempo puerta balón. PERSTEMI= Registro Peruano de Infarto de Miocardio ST elevado.

Lamentablemente no hay estudios poblacionales sobre la incidencia del IMCEST en nuestro país. Una aproximación a la forma cómo se ha estado manejando esta entidad clínica se puede derivar de los estudios RENIMA ^9^ y de los registros PERSTEMI I y II realizados en el 2016 y el 2020 respectivamente, siendo estos últimos registros con mayor participación de casos de la ciudad de Lima y de hospitales del Seguro Social EsSalud **(**Figura 4**)**. Del 2016 al 2020 se observa que el porcentaje de pacientes sin tratamiento de reperfusión del infarto se incrementó de 16 ^10^ a 34% ^(^^11^, esto último influenciado por la pandemia de la COVID-19 que complicó notablemente el tratamiento del infarto ^12^. Muchos de estos pacientes no recibieron un tratamiento de reperfusión pues accedieron tardíamente a un centro con capacidad para realizarla (farmacológica o mecánica) básicamente por demoras del propio sistema ya que la demora del paciente (tiempo a primer contacto médico) se mantuvo en este periodo en una mediana de aproximadamente 2 h. Finalmente, lo anterior se ve reflejado en que solo la mitad de los casos de IMCEST acceden a una reperfusión oportuna (< 12 h). La fibrinolisis sigue siendo el principal esquema de tratamiento del IMCEST en alrededor del 40% de casos ^10^^,^^11^, cabe destacar el aumento de casos con estrategia farmacoinvasiva en los últimos años, algo que es de suma importancia ya que se vislumbra como la mejor estrategia de manejo dadas las condiciones de nuestro sistema de salud, accesibilidad al tratamiento y de las condiciones geográficas en nuestro país que hace difícil que se llegue a tiempo para una intervención coronaria percutánea primaria (ICPp).

La ICPp, realizada en su mayoría en unos pocos centros de Lima, se administra aproximadamente a la tercera parte de los casos ^11^, por debajo de lo encontrado en un registro de Argentina (89%) ^13^, similar a lo encontrado en Brasil (29,7%) ^(^^14^ y mayor a lo encontrado en México (15%) ^15^.

Los problemas en el acceso oportuno a centros con capacidad de diagnóstico y tratamiento adecuado generan tiempos de transferencia de hasta 3,5 h hacia centros con capacidad de intervencionismo ^11^, lo que hace necesaria la creación de «redes de infarto» que articulen los esfuerzos entre los diferentes subsistemas de salud, lo que no solo implica la logística o la infraestructura hospitalaria, sino también la capacitación constante del personal de salud y de la población en general.

### Importancia de las redes de infarto

Durante las últimas dos décadas, la implementación de redes de atención para el tratamiento del IMCEST han mostrado disminuir los tiempos de atención, aumentar las estrategias de reperfusión, disminuir la mortalidad y aumentar las tasas de ICPp en estos pacientes ^(^^16^^,^^17^. Este enfoque requiere mejoras en el sistema de salud y coordinación multidisciplinaria, abarcando la educación pública sobre el reconocimiento de los síntomas del IMCEST, la atención prehospitalaria, la comunicación fluida entre servicios de emergencia, cardiología y centros con intervención coronaria percutánea (ICP), e incluso la colaboración interinstitucional cuando sea necesario ^(^^18^.

La creación de cada red de atención de IMCEST implica una supervisión continua del perfil demográfico de la región y la creación de vínculos de comunicación entre centros de atención de diversos niveles de complejidad. Uno de los métodos es la utilización de la telemedicina (WhatsApp, Apps *ad hoc*, Webs) que han sido probados con efectividad, incluso en países de bajo o mediano ingreso ^19^^,^^20^. Esto permite adaptar la estrategia de reperfusión a las condiciones específicas de cada área geográfica, garantizando la eficiencia en el tiempo.

En Perú, la mayoría de los pacientes con IMCEST ingresan a centros que carecen de capacidad para realizar intervenciones coronarias percutáneas, lo que hace que la estrategia farmacoinvasiva sea una opción viable debido a los retrasos en el diagnóstico y transporte ^(^^11^. No obstante, es crucial unificar la atención de estos pacientes, lo que requiere un mayor apoyo de las autoridades en las instituciones de salud, el personal médico, las redes de comunicación y la comunidad, para mejorar la atención y reducir la mortalidad por enfermedades cardiovasculares a largo plazo.

En la Tabla 3, se detallan algunas recomendaciones para la creación de una red local de infarto ^(^^21^^-^^23^.

**Tabla 3:** Recomendaciones para la creación de una red local de infarto ^22^^,^^23^

Recomendación	Actividades
Definición clara de las áreas geográficas de responsabilidad.	Realizar un mapa jurisdiccional señalando las distancias con el centro hospitalario central (hub) y los centros periféricos (spokes) que ofrezcan emergencia 12 o 24 h.
	Establecer un diagnóstico situacional de la capacidad resolutiva de cada centro para la atención del IMCEST (disponibilidad de electrocardiograma, fibrinolisis, medicación coadyuvante, etc.).
	Tener un teléfono único y utilizar la herramienta de WhatsApp para una comunicación efectiva entre el *hub* y los spokes a fin de poder realizar las coordinaciones de los pacientes.
Protocolos compartidos, basados en la estratificación de riesgos y el transporte por personal médico, enfermera o equipo de paramédicos capacitados en vehículos de transporte adecuadamente equipados.	Contextualizar las guías internacionales y nacionales de manejo del IMCEST a protocolos locales señalando el flujograma de los pacientes desde el ingreso por dolor torácico hasta las responsabilidades del personal de salud que trata al paciente.
	Crear comités locales de respuesta ante un IMCEST comprometiendo a las autoridades en el hospital, los servicios de emergencia y cardiología.
Clasificación prehospitalaria de pacientes con un diagnóstico de trabajo de IMCEST, de acuerdo con la estratificación de riesgo (electrocardiograma y síntomas), hacia el centro apropiado, evitando hospitales sin capacidad de reperfusión o sin servicio las 24 h.	De ser el caso se debe coordinar con los servicios de ambulancias para una vez que se diagnostique un IMCEST sea transportado a hospitales que, al menos, tengan posibilidad de realizar una fibrinolisis.
	Al llegar al hospital con capacidad para ICPp el paciente con sospecha de IMCEST debe ser llevado inmediatamente al laboratorio de cateterización, evitando el área de emergencia y otras áreas de hospitalización.
	Si el diagnóstico de trabajo de IMCEST no ha sido realizado por el equipo de la ambulancia y la ambulancia llega a un centro sin disponibilidad reperfusión, la ambulancia debe esperar el diagnóstico y, si se realiza un diagnóstico de trabajo de IMCEST, debe continuar hacia un hospital capacitado para reperfusión.
Los pacientes que llegan a un hospital no capacitado para ICP y esperan transporte para ICPp/o de rescate deben ser atendidos en un área adecuadamente monitoreada y con personal.	Las áreas de emergencia deben de adecuar en la unidad de shock trauma los recursos necesarios para la atención de pacientes con IMACEST (coche de paro, monitor cardiaco, línea de oxígeno, entre otros).

ICP= intervención coronaria percutánea. IMCEST= infarto de miocardio con elevación del ST.

## Consideraciones generales de manejo del IMCEST no complicado

El IMCEST no complicado es aquel que se presenta sin signos ni síntomas de falla cardiaca aguda, sin complicación mecánica o arritmias importantes. Tras la confirmación electrocardiográfica del SCA con elevación del segmento ST, se debe activar el servicio de emergencias, de acuerdo con cada escenario individual del centro de primer contacto. Dentro de las medidas generales de manejo se recomienda ^4^^,^^6^^,^^24^:

- Colocar al paciente en reposo absoluto con elevación de la cabeza y, si fuera posible, pesarlo previamente.

- Monitoreo de signos vitales: presión arterial, frecuencia cardiaca y saturación de oxígeno. Monitorización de presión arterial no invasiva cada 5 min, monitoreo con posibilidad de desfibrilación o cardioversión eléctrica. 

- Canalizar al menos dos vías venosas periféricas con Abbocath número 16 G o 18 G , evitando la flexura del codo (vía antecubital), en el antebrazo izquierdo de preferencia. Evitar colocar una vía endovenosa central, la vía intramuscular y la colocación de sonda Foley. 

- Extracción de sangre para determinación de exámenes de rutina de laboratorio, sin que esto retrase el inicio del tratamiento o el traslado. A todos los pacientes se sugiere solicitar hemograma, glucosa, urea, creatinina, perfil hepático, perfil lipídico, perfil de coagulación, hemoglobina glicosilada, grupo sanguíneo y factor RH, troponina, CPK - MB (en ausencia de troponina).

La estrategia de reperfusión a elegir en cada paciente dependerá del tiempo de evolución de los síntomas, la disponibilidad de recursos de cada centro o red, y los periodos de tiempos estimados a la reperfusión.

**Figura 5:**
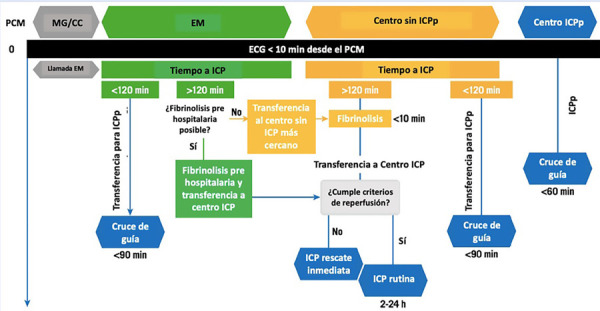
Estrategias de reperfusión recomendadas de acuerdo con el tiempo y el punto de entrada a la red ^26^. ECG= electro cardiograma. PCM= primer contacto médico. EM= servicios de emergencias médicas. MG/CC= médico general/ cardiólogo clínico. ICP= intervención coronaria percutánea. ICPp= intervención coronaria percutánea primaria.

En pacientes con menos de 12 h de evolución desde el inicio de los síntomas, la máxima demora prevista entre el diagnóstico del IMCEST y la ICPp que se recomienda para escoger entre la estrategia de ICPp o la fibrinolisis, es de un tiempo menor/igual a 120 min **(**figura 5**)**^6^^,^^19^^,^^24^^-^^26^.

Para cumplir este objetivo de 120 min, desde un centro sin capacidad de ICPp, la duración entre el arribo del paciente y su salida en ambulancia en dirección hacia el centro con capacidad de ICPp debería ser menor a 30 min. Si el centro sin ICPp está localizado a menos de 30 min de tiempo de distancia del centro con ICPp y este último está listo para aceptar e ingresar al paciente a sala de hemodinámica (tiempo puerta - balón del centro con ICPp objetivo menor/igual a 60 min), se debe trasladar al paciente hacia el centro con ICPp.

 Si el centro sin ICPp está localizado a más de 30 min de distancia del centro con ICPp, este plazo de 120 min no se podrá cumplir, por lo que se sugiere que estos pacientes deban ser tratados con estrategia farmacoinvasiva, es decir, iniciar inmediatamente la fibrinolisis seguida de la referencia para ICP temprana de rutina (dentro de las 2 - 24 h siguientes) si la lisis es exitosa, o ICP de rescate en caso esta sea fallida ^24^^-^^27^.

En caso el centro de primer contacto del paciente no tenga capacidad de ofrecer ninguna terapia de reperfusión, el paciente debe ser inmediatamente trasladado en ambulancia al centro más cercano con capacidad de ofrecerla, ya sea fibrinolisis o ICPp (esta última solo en caso de que el tiempo de traslado hacia el centro con ICPp sea menor a 30 min y el tiempo puerta - balón del centro con capacidad de ICPp sea menor/igual a 60 min). Por otro lado, si el paciente tiene alguna contraindicación para la fibrinolisis, debe ser trasladado para ICPp por cualquier medio ^24^^-^^26^.

Así, la ICPp podría ejecutarse en los tiempos recomendados, fundamentalmente en pacientes localizados cerca de centros con sala de hemodinámica 24/7. En nuestro país, esta es una opción principalmente para áreas urbanas de Lima con tiempo de traslado menor a 30 min hacia centros (públicos o privados) con capacidad de ICPp. Sin embargo, a nivel público en el Perú, solo disponemos de una sala con capacidad de ICPp 24/7, la cual está en la ciudad de Lima y pertenece a EsSalud.

Ante el acceso limitado a una ICPp en tiempos adecuados y teniendo en cuenta que el promedio de tiempo entre el ingreso a un centro con capacidad de ICP y el cruce de la guía es de aproximadamente 70 min **(**figura 4**)**^10^^,^^11^, contamos con aproximadamente 50 min entre el diagnóstico del infarto, la coordinación para transporte y el transporte en sí hasta el centro ICP; por lo que la estrategia farmacoinvasiva debería ser la terapia de reperfusión de elección en la mayor parte de los centros públicos a nivel nacional.

**Figura 6:**
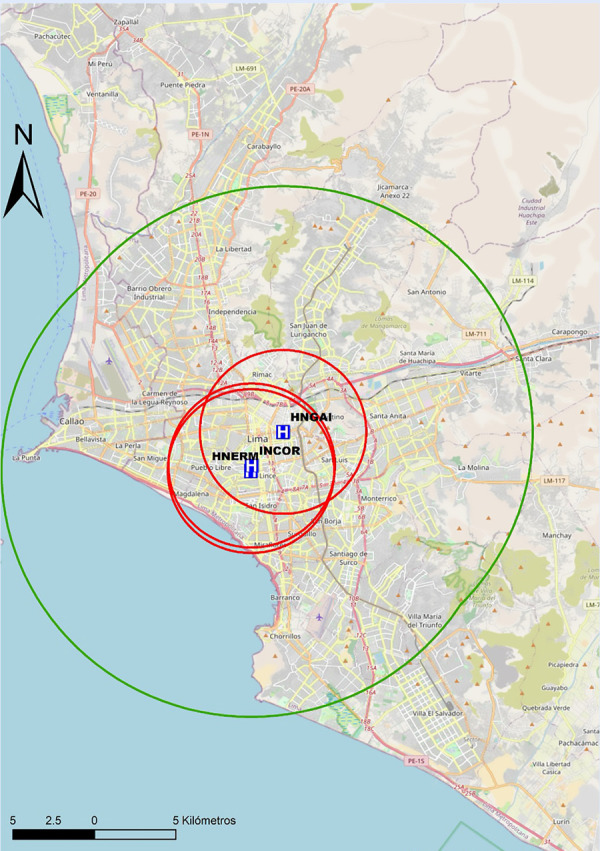
Mapa de distancia a recorrer en 30 min hacia centros con capacidad de ICP, pertenecientes a EsSalud en Lima. ICP = intervención coronaria percutánea. El círculo rojo abarca un radio de 5 km transitables en 30 min en la peor condición del tráfico vehicular estimado. El círculo verde abarca un radio de 15 km transitables en 30 min en la mejor condición del tráfico vehicular estimado. INCOR= Instituto Nacional Cardiovascular. HERM= Hospital Nacional Edgardo Rebagliati Martins. HNGAI= Hospital Nacional Guillermo Almenara Irigoyen.

Recomendamos que cada centro realice un mapeo propio de sus tiempos, para preestablecer y desarrollar protocolos locales de actuación y definir la estrategia de reperfusión más precoz. En las Figuras 6**y**7 se proponen mapas con radio de 30 min de distancia de traslado hacia los centros públicos con sala de hemodinámica en Lima, por lo que se recomienda que centros localizados fuera de este radio de cobertura deberían considerar la estrategia farmacoinvasiva como la de primera elección. Por tanto, para centros públicos en el país sin sala de hemodinámica, o sin capacidad de ICPp en tiempos apropiados, la estrategia por defecto debería ser fibrinolizar y referir (estrategia farmacoinvasiva).

**Figura 7:**
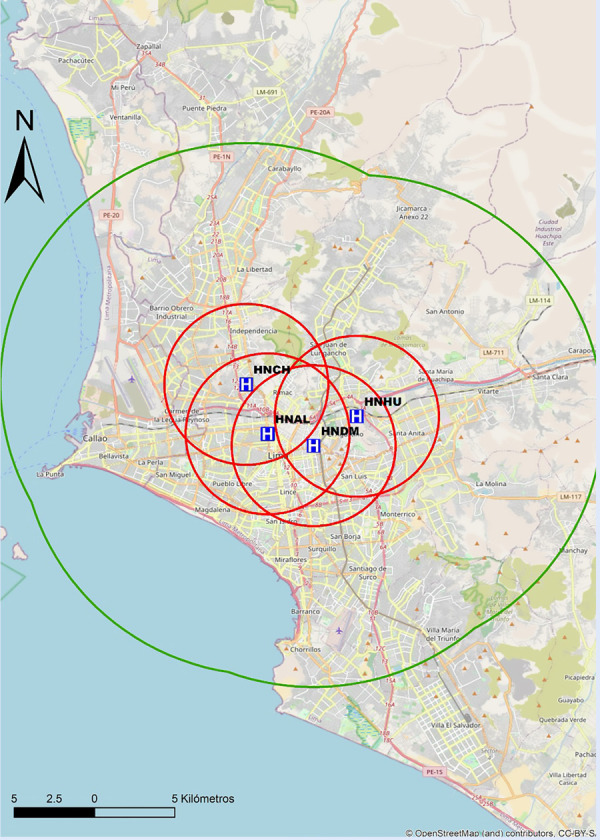
Mapa de distancia a recorrer en 30 min hacia centros con capacidad de ICP, pertenecientes al Ministerio de Salud en Lima. ICP = intervención coronaria percutánea. El círculo rojo abarca un radio de 5 km transitables en 30 min en la peor condición del tráfico vehicular estimado. El círculo verde abarca un radio de 15 km transitables en 30 min en la mejor condición del tráfico vehicular estimado. HNCH= Hospital Nacional Cayetano Heredia. HNAL= Hospital Nacional Arzobispo Loayza. HNDM= Hospital Nacional Dos De Mayo. HNHU= Hospital Hipólito Unanue.

**Figura 8:**
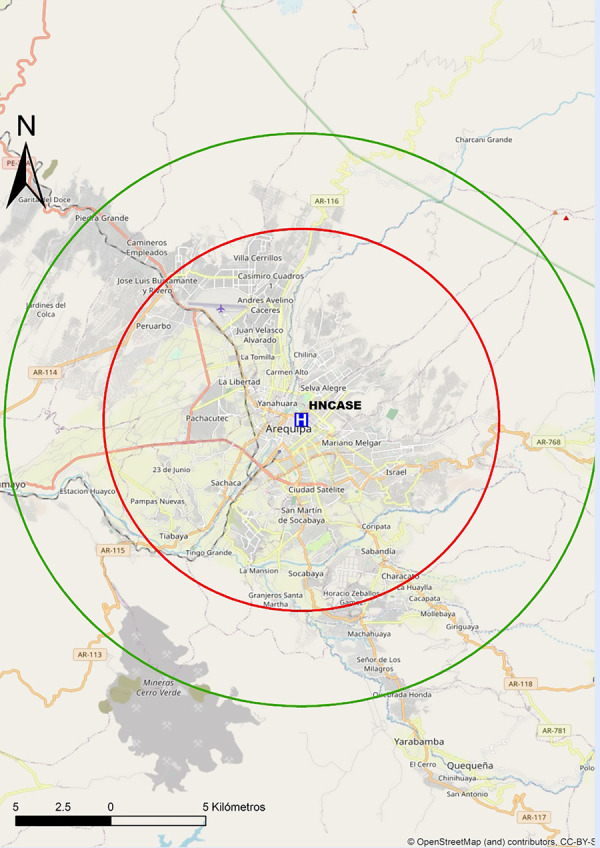
Mapa de distancia a recorrer en 30 min hacia centro con capacidad de ICP en Arequipa. ICP = interven ción coronaria percutánea. El círculo rojo abarca un radio de 10 km transitables en 30 min en la peor condición del tráfico vehicular estimado. El círculo verde abarca un radio de 15 km transitables en 30 min en la mejor condición del tráfico vehicular estimado. HNCASE= Hospital Nacional Carlos Alberto Seguin Escobedo.

**Figura 9:**
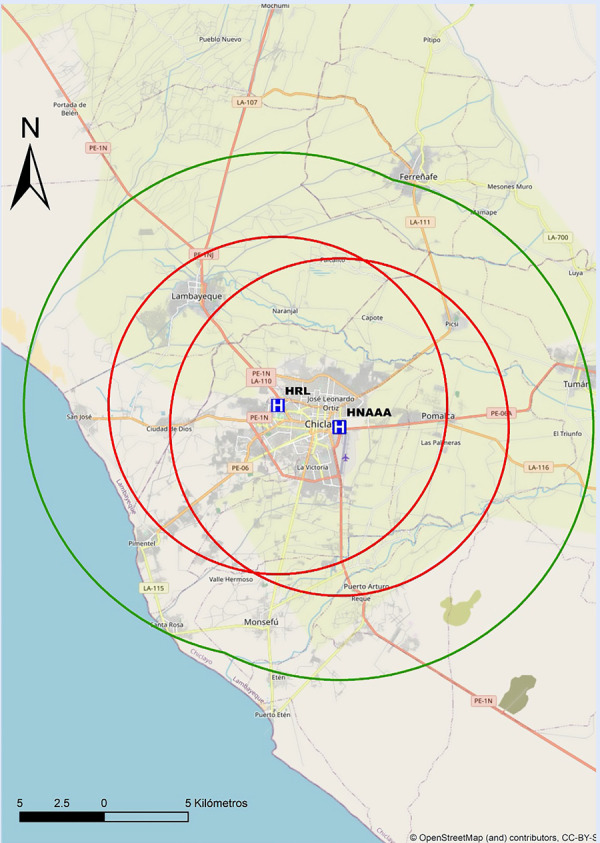
Mapa de distancia a recorrer en 30 min hacia centros con capacidad de ICP en Lambayeque. ICP = interven ción coronaria percutánea. El círculo rojo abarca un radio de 5 km transitables en 30 min en la peor condición del tráfico vehicular estimado. El círculo verde abarca un radio de 15 km transitables en 30 min en la mejor condición del tráfico vehicular estimado. HRL= Hospital Regional Lambayeque. HNAAA= Hospital Nacional Almanzor Aguinaga Asenjo.

Así mismo, en las Figuras 8**,**9**y**10 se adjuntan mapas propuestos con radio de 30 min de distancia de traslado hacia los centros públicos con sala de hemodinámica en Arequipa, Lambayeque y La Libertad.

**Figura 10:**
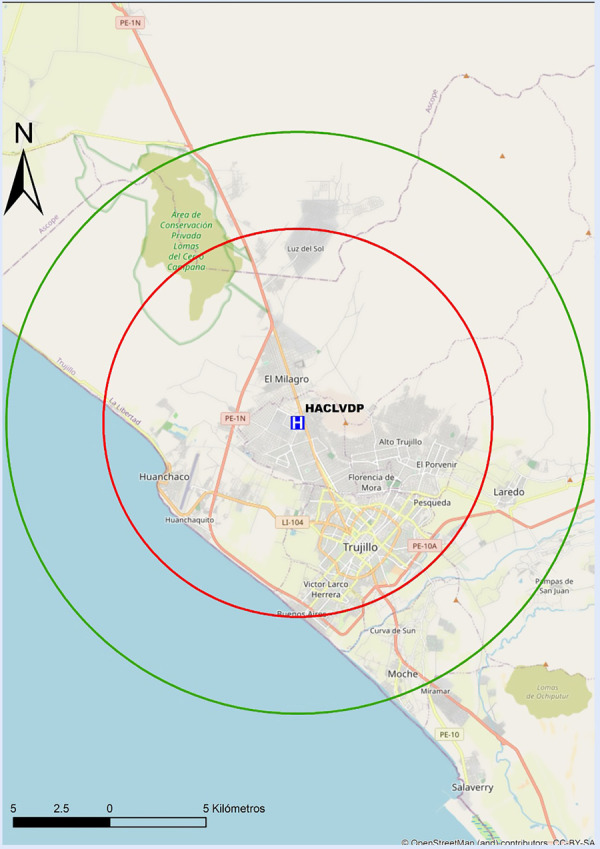
Mapa de distancia a recorrer en 30 min hacia centros con capacidad de ICP en La Libertad. ICP = intervención coronaria percutánea. El círculo rojo abarca un radio de 5 km transitables en 30 min en la peor condición del tráfico vehicular estimado. El círculo verde abarca un radio de 15 km transitables en 30 min en la mejor condición del tráfico vehicular estimado. HACLVDP= Hospital Alta Complejidad Virgen de la Puerta Trujillo.

Para todos los pacientes, después de la administración de la fibrinolisis, está indicado inmediatamente el traslado a un centro con capacidad de ICP. Una vez definido el centro de destino el paciente debe ser transportado por ambulancias con capacidad adecuada para el manejo de pacientes con IMCEST ^24^.

En el hospital con capacidad de ofrecer reperfusión mediante ICP (primaria, de rescate o temprana de rutina), se debe activar el equipo encargado de sala de hemodinámica para llevar a cabo el procedimiento dentro de los plazos de tiempo sugeridos ^6^^,^^21^.

## Paso a paso para la reperfusión farmacológica del IMCEST no complicado

La meta más importante en el manejo del IMCEST es la rápida y completa restauración del flujo sanguíneo en la arteria responsable del infarto (ARI). Para lograr la reperfusión en esta arteria, la terapia farmacológica (fibrinolisis) debe ser instaurada en aquellos pacientes que no tengan una contraindicación para su uso **(**Tabla 4**)**, de forma rápida y completa, ya que el beneficio de esta terapia es tiempo-dependiente, por ende, en cuanto tardemos más en actuar menos será la tasa de reperfusión.

**Tabla 4:** Contraindicaciones a la terapia fibrinolítica ^(^^6^

Contraindicaciones absolutas	Contraindicaciones relativas
Hemorragia intracraneal previa o evento isquémico cerebral de origen desconocido en cualquier momento	Accidente isquémico transitorio en los últimos 6 meses
Evento isquémico cerebral en los últimos 6 meses	Anticoagulación oral
Daño del sistema nervioso central o neoplasia o malformación arteriovenosa	Embarazo o dentro del primer mes posparto
Trauma mayor reciente, cirugía o lesión cerebral (en el pasado mes)	Hipertensión refractaria (PAS >180 mmHg y/o PAD>110 mmHg)
Sangrado gastrointestinal en el pasado mes	Enfermedad hepática avanzada
Discrasia sanguínea conocida (ejemplo: hemofilia, se excluye menstruación)	Endocarditis infecciosa
Disección aórtica	Úlcera péptica activa
Punciones no compresibles en las últimas 24 h (biopsia hepática, punción lumbar)	Resucitación traumática o prolongado (>10 min)

PAS: presión arterial sistólica. PAD: presión arterial diastólica.

Es importante recordar que, si se actúa en las primeras 6 h, el éxito de la fibrinolisis será mayor y la disminución en la mortalidad también será mayor, esto independiente del sexo, la edad, frecuencia cardiaca, presión arterial, historia previa de infarto de miocardio y/o diabetes, a diferencia de si se actúa entre las 6-12 h donde el beneficio disminuye en ~30% ^27^^,^^28^. Este enunciado va en relación con que a mayor tiempo de isquemia el trombo coronario será más rígido, presentará mayor carga de fibrina, y con mayor tiempo de isquemia; asimismo, el paciente presentará mayor riesgo de deterioro hemodinámico que hará, a su vez, menos efectiva la llegada del fármaco a nivel coronario ^29^.

**Figura 11:**
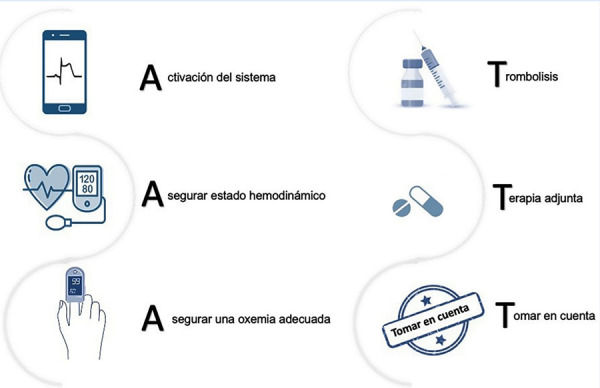
Paso a paso para la reperfusión farmacológica en el IMCEST no complicado (sin shock cardiogénico, ni complica ción mecánica). IMCEST: infarto de miocardio con elevación del ST.

A continuación, detallamos paso a paso las indicaciones que deberían ser tomadas en cuenta durante la reperfusión farmacológica de un paciente con IMCEST no complicado **(**Figura 11**)**, muchas de estas indicaciones pueden darse de forma paralela o consecutiva, por lo que es importante trabajar en equipo con el personal de enfermería:

1. Activación del sistema. Una vez que se identifica al paciente con IMCEST debemos dar aviso al centro con capacidad de ICP más cercano, además de notificar al servicio de ambulancias que se iniciará la fibrinolisis, para que al margen del resultado (previsto tras 90 min de su inicio), se inicie la coordinación de la referencia urgente.

2. Asegurar un adecuado estado hemodinámico. El paciente debe ingresar a una unidad de monitoreo continuo (trauma shock), cerca de un coche de paro para tratar cualquier complicación (arresto cardiaco, taquicardia supra o ventricular). Una vez en esta unidad, enfermería debe hacer el control de funciones vitales, y la canalización de vías periféricas previamente mencionado ^30^. Una vez canalizada la vía se podrá iniciar la administración del fibrinolítico, y posteriormente se pueden sacar muestras para analítica convencional.

- Asegurar una presión arterial sistémica (PAS) >= 90 mmHg y una presión arterial media (PAM) >=65 mmHg que favorezca una adecuada perfusión tisular.
- Si hay hipotensión (PAS < 90 mmHg o PAM <65 mmHg), considerar un IMCEST complicado, requiere un manejo especial, y debería ser notificado al centro con capacidad de ICP para manejo conjunto ^31^.

3. Asegurar una oxemia adecuada. Si la saturación de oxígeno (SatO2) es <90%, se ha de administrar oxígeno en forma de dispositivo de bajo (cánula nasal) o alto flujo (sistema Venturi) para mantener una SatO2 >90% (no es recomendado llevar a una SatO2 de 96%) ^32^^,^^33^.

4. Fibrinolisis. En nuestro medio, debido a su fibrinoespecificidad y disponibilidad, debemos usar alteplase como primera opción, que asociada a la terapia adjunta puede lograr alcanzar una apertura del vaso epicárdico (flujo TIMI 2 o 3) en 73-84% de los casos a los 90 min de haber sido administrado el medicamento ^34^^).^

La preparación de 50 mg viene acompañada de un vial de solución estéril de 50 mL, con lo cual se diluirá (no mezclar vigorosamente) y constituirá una solución de 1mg/mL. Hay que administrarlo de la siguiente forma según el peso (precaución en mujeres, ancianos ≥75 años y en personas de peso <65 kg donde es preferible ser exactos con la dosis en mg/kg para evitar sangrado).

● Si peso <65 kg:

o 15 mg en forma de bolo intravenoso en 2 min, luego

o 0,75 mg/kg en forma de infusión por 30 min (dosis máxima 50 mg), luego

o 0,5 mg/kg en forma de infusión por 60 min (dosis máxima 35 mg).

● Si peso ≥65 kg:

o 15 mg en forma de bolo intravenoso en 2 min, luego

o 50 mg en forma de infusión por 30 min, luego

o 35 mg en forma de infusión por 60 min.

Es importante, posterior a haber acabado la infusión de alteplase, retirar la botella y colgar una solución salina fisiológica de 100 mL para terminar de pasar el remanente que quede en la vía periférica ^35^^,^^36^.

En caso se disponga de Tenecteplase (TNK) como agente fibrinolítico, se administrará como bolo único EV en la dosis de 30 mg (peso < 60 Kg), 35 mg (peso 60 a 69 Kg), 40 mg (peso 70 a 79 Kg), 45 mg (peso 80 a 89 Kg) y 50 mg (peso > 90 Kg). En mayores de 75 años se recomienda reducir la dosis a la mitad ^21^.

5. **Terapia adjunta.** La terapia adjunta está compuesta de doble antiagregación y anticoagulación, con lo que se permite obtener mejores resultados a los 90 min (TIMI 3), disminuyendo así la posibilidad de reoclusión y reinfarto en un 40-50% con respecto a no usarla ^37^^-^^40^.

a) Antiagregación plaquetaria. Se debe utilizar dos antiagregantes y ajustar la dosis en base a la edad.

o Ácido acetilsalicílico: 150-300 mg, vía oral, masticada, de forma inmediata.

o Clopidogrel: En <75 años 300 mg vía oral y en ≥75 años 75 mg vía oral.

b) Anticoagulación. Enoxaparina o heparina no fraccionada. Usar la vía periférica distinta a donde se pasa el fibrinolítico. 

o Heparina no fraccionada

● En caso de fibrinolisis, o en pacientes no reperfundidos: 60 UI/kg EV, dosis máxima de 4000 UI, luego continuar infusión de 12 UI/Kg/h con un máximo de 1.000 UI/h durante 24-48 h. El objetivo de tiempo parcial de tromboplastina activada (TTPA) es 50-70 s o 1,5-2,0 veces el tiempo de control; se monitorizará TTPA a las 3, 6, 12 y 24 h. No requiere ajuste de dosis en caso de enfermedad renal.

● En caso de ICPp: bolo de 70 - 100 UI/kg EV, luego ajustado en sala de hemodinámica según tiempo de coagulación activado (TCA).

o Enoxaparina: completar la primera dosis de enoxaparina preferiblemente dentro de los primeros 30 min de administrado el alteplase ^36^^,^^41^^-^^43^.

● En caso de fibrinolisis o en pacientes no reperfundidos

▪ Si <75 años: bolo de enoxaparina de 30 mg endovenoso seguido de 10 cc de suero salino fisiológico y 15 min después administrar 1 mg/kg de enoxaparina subcutánea cada 12 h (dosis máxima de 100 mg por cada una de las primeras dos administraciones). 

▪ Si ≥ 75 años: no administrar bolo endovenoso, administrar 0,75 mg/kg subcutáneo cada 12 h (dosis máxima de 75 mg por cada una de las primeras dos administraciones). 

● En caso de ICPp: bolo de 0,5 mg/kg EV. 

● En pacientes con enfermedad renal crónica se sugiere: 

▪ Si la tasa de filtrado glomerular es > 30 mL/min, no hace falta ajustar la dosis.

▪ Si la tasa de filtrado glomerular es 15 - 30mL/min, administrar: 

● Si <75 años: bolo de enoxaparina de 30 mg endovenoso seguido de 10 cc de suero salino fisiológico y 15 min después administrar 1 mg/kg subcutáneo cada 24 h (dosis máxima de 100 mg por cada una de las primeras dos administraciones).

● Si ≥ 75 años: no dar bolo, administrar 0,75 mg/kg subcutáneo cada 24 h (dosis máxima de 75 mg por cada una de las primeras dos administraciones).

● Si el paciente se encuentra en diálisis, se recomienda usar heparina no fraccionada, según las dosis previamente mencionadas.

En caso de la administración de fibrinolisis sola o en caso de no recibir ninguna estrategia de reperfusión se debe mantener la anticoagulación hasta 8 días, o hasta el alta hospitalaria. En caso de ICP, se debe suspender tras esta, salvo que exista una alguna indicación adicional para su mantenimiento (por ejemplo, fibrilación auricular, o trombo intracardiaco)

c) Terapia para alivio de síntomas

o Nitratos. En nuestro medio, según disponibilidad, se puede valorar usar dinitrato de isosorbide 5 mg sublingual y repetir hasta 3 veces cada 5 min. Sin embargo, el máximo beneficio se observa con nitroglicerina vía endovenosa en infusión continua, especialmente en hipertensión arterial sistémica descontrolada o en falla cardiaca aguda. Así, en caso de pacientes con IMCEST e insuficiencia cardiaca Killip Kimball (KK) 2-3, se puede usar nitroglicerina endovenosa, diluyendo 2 ampollas de 25 mg/5 mL (50 mg/10 mL) en 240 mL de solución fisiológica y titular en base a la presión arterial, asegurando una PAS>90 mmHg y una PAM ≥ 65 mmHg (titular dosis de nitroglicerina entre 10 mcg/min a 200 mcg/min). Evitar el uso de nitratos si ha habido uso de los inhibidores de la fosfodiesterasa tipo 5 en las últimas 24-48 h (sildenafilo), si hay elevación del segmento ST en el electrocardiograma en V3R - V4R, bradicardia o taquicardia extrema (frecuencia cardiaca <40 o >120 latidos por minuto respectivamente) ^6^^,^^21^.

o Opioides. Se puede usar clorhidrato de morfina de 2-5 mg endovenoso (optar por dosis más bajas 2-3 mg si peso < 60 kg), diluido en 10 mL de suero salino fisiológico, se reevaluará el dolor cada 5 min. El uso de morfina puede generar nauseas las que pueden ser manejadas con metoclopramida 10 mg vía endovenosa.

d) Estatinas. Uso de atorvastatina 40 - 80mg o rosuvastatina 20-40 mg tan pronto como sea posible. Administradas de forma temprana en el contexto de IMCEST han demostrado mejorar el flujo sanguíneo en la ARI, disminuir el daño por isquemia/reperfusión y, por ende, el tamaño del infarto ^44^^-^^46^.

6. Tiempo a resolución y otras consideraciones a tener en cuenta. El éxito de la reperfusión farmacológica lo veremos a los 90 min de iniciada la terapia fibrinolítica. Los criterios de éxito son los siguientes:

a. En el electrocardiograma, la caída ≥ de 50% del segmento ST en la derivada con mayor supradesnivel, presenta la mayor sensibilidad 70-94%, y una especificidad de 54-67%, con un valor predictivo negativo >80% ^47^^,^^48^.

b. Ritmo idioventricular, definido por 3 o más complejos prematuros ventriculares consecutivos a una frecuencia de 40 a 100 lpm, con una sensibilidad de 45% y especificidad de 94%, presentando el valor predictivo positivo más alto, de presentarse en los primeros 90 min ^49^.

c. Resolución de los síntomas, forma parte de los criterios de forma tradicional, pero muchos pacientes, aun con reperfusión exitosa, pueden seguir presentando dolor y este se verá muchas veces influenciado por la cantidad de analgésicos administrados, entre otras causas. De los tres criterios es el de menor seguridad ^50^.

Es importante recordar que durante la fibrinolisis se puede encontrar de forma frecuente contracciones ventriculares prematuras y hasta taquicardia ventricular, y al estar en relación con la reperfusión a nivel microvascular su aparición es autolimitada, bastante bien tolerada e indicador de buen pronóstico (ingreso de calcio intracelular y radicales libres producto de la rápida apertura de la ARI) ^51^.

Teniendo en cuenta todo esto, tenemos que actuar de forma rápida y completa para poder cortar el circuito de isquemia miocárdica y tener el mayor beneficio, que de lejos supera el riesgo de complicaciones. El riesgo de sangrado debe ser evaluado de forma individual (la tasa de sangrado intracraneal es en general de ~ 0,9-1% de los casos, y tasa de sangrado mayor es de ~ 1-1,5% de los casos) ^37^^,^^38^, sopesando el riesgo/beneficio que se tendrá a largo plazo al reperfundir el área isquémica.

En la Tabla 5 se adjunta una lista de chequeo sugerida durante la reperfusión farmacológica del IMCEST.

**Tabla 5:** Lista de chequeo propuesta durante la reperfusión farmacológica del IMCEST

Centro/Hospital:	Número HCl: Nombre y apellidos: Fecha de nacimiento: __/__/___ Sexo: ____Peso:______
1. Confirmar indicaciones para fibrinolisis (tiempo de síntomas, ECG, distancia a centro con ICP)	Fecha y hora de inicio de síntomas: _______ Hora de llegada al hospital (PCM): _______
2. Contraindicaciones para fibrinolisis.	Absolutas: Sí ( ) No ( ) Relativas: Sí ( ) No ( )
3. Activar el sistema/Avisar al sistema de ambulancia Acceso venoso periférico (2) Asegurar estado hemodinámico: Asegurar buena oxemia: ECG:	Abbocath 16 G o 18G PA: ______ FC: ________ FR: ______ SatO2: _____ Basal: ____ 90 min: _____
4. Fibrinolisis	Alteplase: 15 mg e.v. en 2 min, luego 0,75 mg/kg en 30 min y 0,5 mg/kg en 60 min.* DOSIS: _______ TIEMPO: ________
5. Terapia adjunta	Aspirina masticable: 300 mg DOSIS:_____ TIEMPO:_____ Clopidogrel 75 mg o 300 mg* DOSIS: _____ TIEMPO:_____ Anticoagulación: enoxaparina o HNF* DOSIS: _____ TIEMPO: _____
6. Tomar en cuenta	Cumple criterios de éxito de fibrinolisis: (Sí) (No) - Caída del ST ≥ 50% en derivada con mayor supradesnivel - Ritmo ideoventricular acelerado. - Desaparición de síntomas de isquemia.
7. Manejo posfibrinolisis	ECG Contactar con centro con capacidad de ICP y comentar éxito o fracaso de fibrinolisis: traslado no urgente y/o urgente respectivamente. Monitoreo cardiaco continuo.

ECG: electrocardiograma. PA: presión arterial. FC: frecuencia cardiaca. FR: frecuencia respiratoria. SatO2: saturación de oxígeno.

* Valorar dosis de acuerdo a peso, edad, y función renal, para cada medicamento según se especifica a detalle en el texto.

## Casos especiales

### Pacientes con enfermedad renal crónica

Los pacientes con enfermedad renal crónica (tasa de filtración glomerular <60 mL/min/1,73 m^2^) representan hasta un tercio de los síndromes coronarios agudos ^52^. Se evidencia que el pronóstico a corto y largo plazo es peor que en pacientes sin afectación de la función renal, probablemente en relación con la mayor asociación con comorbilidades y complicaciones intrahospitalarias ^53^^,^^54^. En los pacientes sometidos a terapia de reemplazo renal la mortalidad a 30 días alcanza hasta 37,2% ^54^.

Las guías de práctica clínica establecen que si no es posible la referencia para ICPp en menos de 2 h se puede optar por el manejo fibrinolítico; sin embargo, es escasa la información respecto a fibrinolisis en IMCEST en pacientes con enfermedad renal crónica, pues estos con frecuencia son excluidos de los ensayos clínicos, y las guías de práctica clínica no aportan una estrategia definida ^21^. Esto ha hecho que la terapia fibrinolítica sea poco utilizada en pacientes con disfunción renal lo cual contribuye al mal pronóstico mencionado ^53^.

Es necesario generar información basada en evidencia para asistir a los clínicos en el manejo fibrinolítico de estos pacientes de alto riesgo isquémico y de sangrado; sin embargo, la ausencia de ensayos clínicos no debe privar al paciente de la terapia de reperfusión farmacológica. En el caso de terapia fibrinolítica debe asegurase la reducción de factores de riesgo de sangrado modificables, como ajustar la dosis de la anticoagulación según función renal ^21^^,^^54^.

### Adultos mayores

La morbimortalidad de los pacientes con IMCEST aumenta con el incremento de la edad ^55^. En caso de dificultad de acceder a ICPp dentro de las 2 h del primer contacto médico, se plantea la estrategia farmacoinvasiva como una excelente alternativa de manejo. En el estudio STREAM-1 se demostró que los pacientes con IMCEST que se presentaron dentro de las 3 h del inicio del dolor, que no podían acceder a ICPp en primera hora, y que recibieron la estrategia farmacoinvasiva, presentaron tasas similares de muerte, *shock*, falla cardíaca o reinfarto comparado con la ICPp ^56^. Se observó un exceso de hemorragia intracraneal en pacientes =>75 años, por lo que se redujo la dosis de fibrinolítico a la mitad, sin episodios subsecuentes de sangrado intracraneal. 

Ante este hallazgo, el estudio STREAM-2 se diseñó para evaluar la eficacia y seguridad de mitad de dosis de tenecteplase en adultos =>60 años con IMCEST. Los puntos finales de eficacia (resolución del segmento ST y muerte, *shock*, falla cardíaca o reinfarto a 30 días) fueron similares en ambos grupos. Hubo mayor número de hemorragias intracraneales en el grupo farmacoinvasivo, aunque ninguno de estos episodios se produjo en <75 años ^57^. Ante estos hallazgos se recomienda reducir la dosis de tenecteplase a la mitad en pacientes con IMCEST =>75 años ^21^. En ese mismo sentido, un régimen reducido de alteplase podría usarse en >= 75 años de acuerdo con la opinión de expertos, sin embargo, faltan datos contundentes que sugieran el uso de esta dosis en dicha población.

### Pacientes anticoagulados

Entre 6-8% de los pacientes en quienes se realiza ICP tienen indicación de anticoagulación ^21^. Asimismo, la coexistencia de síndrome coronario agudo y fibrilación auricular, con necesidad de anticoagulación varía entre 6-23% ^58^. En este escenario la anticoagulación es una contraindicación relativa para la fibrinolisis ^59^. Se recomienda el traslado a un centro con capacidad de realizar ICPp. Si no es posible, se sugiere analizar detenidamente el riesgo de sangrado para proceder con la fibrinolisis, siendo contraindicada la aplicación de esta terapia con un tiempo de protrombina (INR) >1,7 ^60^. En el caso de anticoagulantes no antagonistas de la vitamina K se recomienda la determinación de su concentración sérica ^59^; sin embargo, no contamos con esa posibilidad en nuestro medio, por lo que podría verificarse el tiempo que se suspendió el medicamento, se requiere > 48 h de suspensión para validar el uso de fibrinolisis.

### Pacientes jóvenes

La delimitación de este grupo es importante debido a que en este grupo de pacientes son menos comunes los factores de riesgo cardiovascular comparativamente a pacientes de mayor edad. El infarto agudo de miocardio (IAM) en jóvenes, en general, puede estar asociado a diferentes mecanismos fisiopatológicos: 1) IAM relacionado a factores de riesgo cardiovascular tradicionales; 2) IAM asociado a drogas; 3) IAM asociado a disección coronaria espontánea; miocarditis o embolismo; 4) IAM asociado a enfermedad ateromatosa (erosión de placa) y 5) vasoespasmo coronario ^61^. En el escenario del IMCEST, se sugiere mantener las mismas recomendaciones de manejo antes mencionadas ^62^. Según los hallazgos del cateterismo cardiaco y el factor etiológico del infarto, se realizarán diferentes estrategias terapéuticas, como angioplastia coronaria percutánea si hay enfermedad ateromatosa; manejo médico o revascularización selectiva en caso de disección coronaria espontánea; estabilización hemodinámica, ampliación diagnóstica y manejo médico en caso de miocarditis; anticoagulación en caso de embolismo, entre otras ^21^.

### Pacientes con sangrado posfibrinolisis

El sangrado posfibrinolisis en pacientes con IMCEST puede presentarse en las primeras 24 h siguientes a su uso, aumenta el riesgo de morbilidad y mortalidad, y se clasifica en sangrado mayor o menor. El sangrado intracraneal es el sangrado mayor más frecuente y preocupante, la fibrinolisis se asocia a mayor sangrado intracraneal en comparación a pacientes que van a ICP.

Existen algunas estrategias para intentar reducir el sangrado post fibrinolisis como: control de la presión arterial en urgencias, ajustar la dosis de los fibrinolíticos, antiagregantes y anticoagulante en base a peso y edad, uso del acceso radial, implante de *stents* modernos, y revascularización completa funcional en enfermedad coronaria multiarterial, entre otras ^21^^,^^63^.

En todo paciente con IMCEST se debe ser valorar el riesgo de sangrado (escala Crusade), y en caso de presentarse, su severidad debe ser estratificada (escala TIMI). En presencia de hemorragia menor generalmente no se requiere algún tratamiento específico ni interrumpir el uso de los medicamentos, a excepción de que la hemorragia sea persistente, en donde debe valorarse suspender la terapia antiagregante y anticoagulante hasta asegurar que no haya sangrado activo. En caso de hemorragia mayor se recomienda, en general, suspender la anticoagulación y la terapia antiagregante, aportar solución salina fisiológica, coloides o plasma fresco congelado, evaluar la necesidad de transfusión de glóbulos rojos, revertir la acción de los fármacos, de ser posible, y si el sangrado es vertiginoso y ante la presencia de inestabilidad hemodinámica persistente evaluar una intervención quirúrgica o endovascular urgente para detenerlo ^64^.

En el caso particular de que se presente un sangrado intracraneal se sugiere:

- Suspender la infusión del fibrinolítico.
- Obtener muestras para hemograma, INR, TTPA, fibrinógeno, tipificación sanguínea, y pruebas cruzadas.
- Una tomografía no contrastada de emergencia, para la valorar extensión y pronóstico.
- Crioprecipitado 10 U endovenoso, durante 10 a 30 min.
- Ácido tranexámico 1000 mg endovenoso, durante 10 min, o ácido 6-aminocaproico 4 - 5 gramos durante 1 h, seguido por 1 g endovenoso hasta el control del sangrado.
- Valorar la administración de una aféresis o seis concentrados plaquetarios, sulfato de protamina para revertir la heparina (1 mg de protamina neutraliza 100 U de heparina), y plasma fresco congelado.
- Evaluar la necesidad de transfusión de glóbulos rojos, en especial si la hemoglobina es menor a 8 gr/dL o el hematocrito es menor a 25%.
-Evaluación por neurocirugía y hematología.
- Terapia de soporte, manteniendo la estabilidad hemodinámica y control de la vía aérea, incluye manejo de la presión arterial, glucosa, temperatura, presión de perfusión cerebral, y presión intracraneal ^64^^,^^65^.

En conclusión, el manejo adecuado del IMCEST implica un diagnóstico certero, la aplicación adecuada de estrategias de reperfusión farmacológica y/o la referencia oportuna a centros de mayor complejidad respetando los tiempos sugeridos en las guías; para lo cual, es imprescindible el trabajo bajo redes de reperfusión. 

## Fuente de financiamiento:

Autofinanciado
